# Tranexamic Acid in a Case Report of Life-threatening Nontraumatic Hemorrhage in Immune Thrombocytopenic Purpura

**DOI:** 10.5811/cpcem.2020.5.46955

**Published:** 2020-07-09

**Authors:** Melanie M. Randall, Jason Nurse, Karan P. Singh

**Affiliations:** * Loma Linda University Medical Center and Children’s Hospital, Department of Emergency Medicine, Loma Linda, California; † San Gorgonio Memorial Hospital, Department of Emergency Medicine, Banning, California

**Keywords:** immune thrombocytopenic purpura, tranexamic acid, epistaxis

## Abstract

**Introduction:**

Immune thrombocytopenic purpura (ITP) is an autoimmune-mediated disorder in which the body produces antibodies that destroy platelets, causing an increased risk of bleeding and bruising. Tranexamic acid (TXA) is a medication that prevents clot breakdown and is used to treat uncontrolled bleeding.

**Case Report:**

We present the case of an 11-year-old female with significant epistaxis and hypotension in the emergency department. Traditional therapies were initiated; however, the patient continued to have bleeding and remained hypotensive, so intravenous TXA was given. The patient’s bleeding then resolved.

**Conclusion:**

TXA may be a safe and effective adjunct to traditional therapies for the treatment of life-threatening hemorrhage in ITP patients.

## INTRODUCTION

Immune thrombocytopenic purpura (ITP) is an autoimmune-mediated disorder in which antibodies to platelets cause a precipitous drop in platelet level. This increases bruising and risk of bleeding. It can be acute, chronic, or recurrent. While most patients do not experience severe consequences, ITP occasionally causes life-threatening hemorrhage.^1^ Tranexamic acid (TXA) is a medication that prevents breakdown of formed clots and is commonly used for traumatic hemorrhage and perioperative bleeding. To date, it has not been described in the setting of life-threatening bleeding secondary to ITP in children. We present a novel case of an 11-year-old female with recurrent epistaxis, hematemesis, and hypotension who was given, along with standard ITP therapies, intravenous (IV) TXA in the emergency department (ED).

## CASE REPORT

An 11-year-old female weighing 62 kilograms with no past medical history presented to the ED by ambulance after two syncopal episodes and recurrent epistaxis. The epistaxis had begun the night before and was associated with multiple episodes of hematemesis. Her measured vital signs by paramedics were a heart rate of 150 beats per minute and blood pressure 76 systolic over 41 diastolic millimeters of mercury. Her physical exam was a general appearance of alert and awake, warm and dry skin, dried blood in the nares, tachycardia with no murmur, clear lung sounds, and a petechial rash on bilateral lower extremities.

The patient was initially given a one liter bolus of lactated Ringer’s solution. A type and crossmatch for packed red blood cell (PRBC) transfusion was sent to the laboratory along with a complete blood count, coagulation studies, and electrolytes. The laboratory results showed a platelet count of 1000 per cubic millimeter (mm3) (reference range [Ref]: 150,000–450,000/mm3); hemoglobin of 7.3 grams per deciliter (g/dL) (Ref: 12.0–16.0 g/dL); hematocrit of 20.9% (Ref: 37.0–47.0%); partial thromboplastin time of 29.6 seconds (Ref: 22.0–34.0 seconds); prothrombin time of 12.1 seconds (Ref: 9.0–12.0 seconds); and international normalized ratio of 1.1 (Ref: 0.8–1.2).

Approximately two and half hours after arrival, the patient had another epistaxis episode with posterior bleeding and 400 milliliters of hematemesis. The patient was given IV dexamethasone 10 milligrams and IV immune globulin (IVIG) of 45 grams (g). The correct dose of IVIG would have been 62 g; however, the pharmacy had only 45 g available at the time. Despite these treatments, the patient remained hypotensive with a systolic blood pressure in the 60s and a heart rate in the 160s.

Nasal packing topically soaked with TXA was placed in the patient’s left nare, and PRBC transfusion was begun; however, due to continued hypotension and instability of the patient, the decision was made to give TXA 1 gram intravenously. Ten minutes after the administration of TXA, the patient’s hematemesis and epistaxis resolved. The patient’s systolic blood pressure improved to the 80s, and her heart rate to the 110s. Platelet transfusion had been ordered but was not available readily, by which time the patient’s vital signs had improved and bleeding had stopped. The patient’s clinical course is noted in the table.

Patient was transferred to a nearby, contracted facility during which she received evaluation for new-onset ITP. She did well and was discharged home without further episodes of serious bleeding.

**CPC-EM Capsule**

What do we already know about this clinical entity?

Immune thrombocytopenic purpura (ITP) can develop acutely, with a risk of bleeding and requiring emergent treatment. Tranexamic acid (TXA) is a medication used to treat hemorrhage in a variety of settings.

What makes this presentation of disease reportable?

The use of intravenous TXA has not been previously described as a treatment for hypotension and hemorrhage control in ITP.

What is the major learning point?

TXA may be a safe and effective adjunct to traditional therapies for the treatment of life-threatening hemorrhage in patients with Immune thrombocytopenic purpura ITP.

How might this improve emergency medicine practice?

Emergency physicians can add this to the list of potential treatments for severe acute hemorrhage in the setting of ITP.

## DISCUSSION

We report a case of life-threatening hemorrhage secondary to acute ITP treated with IV TXA. To our knowledge, this is the first case of this use of TXA reported in the literature. Acute ITP often presents to the ED with easy bruising and mucosal bleeding. In children it can develop suddenly and is often the result of an autoimmune antibody response to platelets.^2^ The incidence of serious hemorrhage is rare, but is highest in patients with very low platelet counts and those who do not achieve remission.^1^,^2^ A standard treatment regimen for pediatric acute ITP includes corticosteroids and IVIG to increase platelet counts; however, another school of thought is to only provide supportive care as most patients will recover without treatment.^1^ Cases refractory to standard treatments have been successfully treated with rituximab, a monoclonal antibody. Newer thrombopoietin receptor antagonists (TPO-RAs) such as eltrombopag and romiplatim, which stimulate bone marrow, have been used in treating chronic ITP.^3^ For acute cases of bleeding, high-dose IV steroids, IVIG, aminocaproic acid, recombinant factor VIIIa, and TPO-RAs have been studied.^1^,^2^ And while TXA has been studied in adults, with anecdotal evidence of its use in controlling mucosal bleeding, and a case series describing its use of TXA in chronic ITP-related bleeding, there are no studies in children.^4^–^6^

When reviewing the literature, we found that TXA has been studied and used in a wide range of settings, especially in the surgical fields including orthopedic procedures, otolaryngologic surgery, obstetric bleeding and heavy menstrual bleeding, pediatric cardiac surgery, and coronary artery surgery.^2^,^8^–^11^ A large meta-analysis evaluating TXA in surgical patients showed that TXA reduced the need for blood transfusion and resulted in fewer deaths.^11^ It has also been studied for the treatment of epistaxis.^10^,^12^ TXA is quickly becoming a standard of care in the treatment of adult trauma patients, with studies ongoing in pediatric trauma patients.^13^ A few of these studies have shown a slightly higher risk for postoperative seizures; however, the majority of studies show that TXA has minimal side effects.^2^, ^9^, ^11^

TXA, which is a synthetic derivative of lysine, acts as an antifibrinolytic by preventing plasminogen from being converted to plasmin. Plasmin breaks down fibrin of already-formed blood clots.^14^ The evidence for antifibrinolytics in hematology patients is limited, but it has been used to treat bleeding related to a variety of hematologic conditions including hemophilia and von Willebrand disease.^2^, ^15^ While there is no evidence that shows TXA should be used in the absence of other traditional treatments for ITP, this case demonstrates its use as an adjunct for unstable bleeding and the need for further study and investigation of TXA use in patients with ITP.

## CONCLUSION

TXA may be a safe and effective adjunct to traditional therapies for the treatment of life-threatening hemorrhage in ITP patients.

## Figures and Tables

**Table:** Timeline of patient care.

Time	Patient course
0827	Patient arrived by ambulance VS: BP 76/41, HR 150, RR 18, SaO_2_ 93% on RA
0900	LR 1L IV bolus
1000	Dexamethasone 10mg IV VS: BP 84/52, HR 123, RR 22, SaO_2_ 99%
1100	IVIG 45gm
1115	Epistaxis resumes and draining posteriorly, Emesis 400mL blood VS: BP 70/40, HR 140, RR 20, SaO_2_ 100% Nasal tampon soaked in TXA inserted
1130	PRBC 1 unit IV started
1134	TXA 1gm IV
1145	Patient reports no further bleeding
1155	VS: BP 82/58, HR 110, RR 22, SaO_2_ 100%

*VS*, vital signs; *BP*, blood pressure; *HR*, heart rate; *RR*, respiratory rate;*SaO_2_*, oxygen saturation; *RA*, room air; *LR*, lactated Ringer’s; *IV*, intravenous; *gm*, gram; *IVIG*, intravenous immunoglobulin; *TXA*, tranexamic acid; *PRBC*, packed red blood cells.
